# Nurses Want a Career

**Published:** 1921-04-09

**Authors:** 


### NURSES WANT A CAREER.

Among the various subjects that engage the attention of recognised leaders of nurses from time to time, it always appears to me that least of all is devoted to the most vitally important—the nurse's career. Nurses' pay is discussed in a sporadic manner—so are their hours of duty, and now the question of education before probation and after is looming. Most useful and necessary are these and kindred subjects, such as the Nation's Fund for Nurses. But the subject of a career to which these are subsidiary seems by common consent to be avoided. This is probably not deliberate. On the contrary, it is very likely that if the leaders referred to give the subject any thought at all they think that by process of patchwork a career may be evolved.

### Working for Posterity.

Of course it may. A whole is made up of many parts. But the process will be so slow that none of us can hope to see it. Like Registration, it may be in course of preparation for practical purposes some time in the next generation. I do not see, however, why all the efforts of the nurses' leaders should be in the interests of posterity. Nurses have accomplished a great deal for this generation, and posterity very little! Does it not seem not only an act of righteousness, but of expediency, that a vigorous effort should be made right now to give the nurse a place in the sun?

### What is a Career?

Somebody will doubtless inquire at this point, what is a career? Well, my conception of a career is something very far in advance of a competence, and until such is forthcoming I am afraid that the faithful chronicler will testify to the continued decline of the profession. The modern girl is advancing with rapid strides, and, even if she does not realise it, she is being taught whither to turn her feet in her search for a field of activity suitable to her acquirements. The nursing field she finds to be more or less a desert. The work is trying and employment precarious. There are few prizes and no pensions to look forward to.

### Nurses at Fault?

I have made it my business to inquire from some of the leaders concerning these matters, and I am informed, truly or otherwise, that a great deal of the fault lies with nurses themselves, and that they positively cannot be got to take a real interest in their own welfare. Thus, apparently, it happens that each nurse refuses to be drawn into any activity on behalf of her profession, and is content to live a hand-to-mouth existence.

I confess that the explanation is not convincing. It may be, and doubtless is, quite true that it is difficult to arouse nurses to a sense of corporate responsibility. So far as I know very little effort has been made in sounding the call. But even if they flatly refuse to be interested the story does not therefore terminate. It is of great and pressing importance to the entire community that a plentiful and efficient nursing supply should be available, and this can be made possible only by allurement. It cannot be accomplished by missionary work. You may preach, but your words will fall on deaf ears. The girl whose service is desired suffers from the belief that she can be useful in some more profitable calling, and her reasoning powers are sufficiently acute to enable her to argue with herself, or with you, that if nursing is important service it ought to be remunerated. I have seen hundreds of references to nursing and to voluntary hospitals of late in the daily press, and sometimes in Parliament, but I cannot recall one where this side of the subject is touched. It seems to be taken for granted that a sufficient number of nurses will always be found at whatever remuneration and on whatever conditions employers may offer. Either this, or the subject is postponed to a more convenient season. Both policies are bound to react with disastrous consequences, and are unfair both to nurses and to the nation.

The subject is one which calls for the serious attention of the ablest and most influential friends of the nursing profession. It concerns hospital governors everywhere, and especially outside the Metropolis, where neglect is not so apparent. But it is of supreme importance to the Minister of Health, whose activities must be seriously impeded if the supply of efficient nurses should become shortened.

### Nurses and Trade Unions.

Those who are responsible should bear in mind that at their request nurses as a body have held aloof from Trade Unions. Their so doing is a proof of their magnificent loyalty to high traditions. It is, however, also a signal of trust reposed in their leaders, whose duty it becomes to ensure their well being. To accomplish this merely requires intelligent activity. The nation is always ready to respond, provided that the facts are fully and fairly represented. *These facts have never been put before the public.* There has been propaganda here a little and there a little, sometimes about this, and sometimes about that. The needs of hospitals have received considerable attention. But the necessity for making a career for civil nurses, as for Army nurses, has never been preached.

It does not appear an adequate explanation that nurses themselves hold aloof from pushing their claims. They have neither the time nor the energy for organised agitation. But if their co-operation is claimed to help forward any movement made on their behalf it will not be found lacking, and twenty thousand blended notes will not sound in vain. IERNE.

